# Extra Limites

**Published:** 1821-09-01

**Authors:** 


### EXTRA LIMITES.

At the annual general meeting of the associated apothecaries and surgeon-apothecaries of England and Wales, held by public advertisement, at the Crown and Anchor Tavern, Strand, July 4, 1821—Joseph Hays, Esq. President;

The following Report from the General Committee was read, viz.—

In conformity with its duty to the Association, and with the laudable custom that has hitherto prevailed, the Committee respectfully submits to the general meeting its annual Report.

It would have been extremely gratifying to all its members could your committee have announced the attainment of that important object which has been so long, and so ardently desired, viz. a legislative provision against the mal-practices of illiterate individuals, who, under usurped titles, carry on the practice of medicine equally to the dishonour of a liberal profession, and to the injury of the community at large. But the Committee still feels that neither House of Parliament is sufficiently aware of the evils which result from the present state of medical practice, as to be convinced of the necessity for a change. It can only regret, therefore, that during the last year no favourable opportunity has presented itself of making a further application to the Legislature.

Parliament is very wisely jealous of making enactments at the suggestions of petitioners, who may be actuated by motives of private advantage, rather than by a desire to benefit the public, and it is not to be expected, the House of Commons having rejected the surgeons' bill on grounds which seem to prove their unacquaintance with the real merits of the question, that Parliament will be at present disposed to grant any effectual relief for the imperfections of the apothecaries' act; nevertheless, your committee is firmly persuaded that in no long time, through the measures adopted, such a mass of evidence will be collected and arranged as will convince the public of the mischiefs and mortalities that are continually happening, from confiding the treatment of difficult, and even dangerous, diseases to ignorant and uneducated persons; and of the absolute necessity thence arising, of resorting to legislative precautions as the only adequate remedies.

Convinced that such is the true state of the case, your Committee has exerted itself to collect evidence at once strictly correct, clear, and decisive; and, in obedience to the 16th and 17th resolutions of the last annual meeting, circular letters have been sent into every quarter in which the Society has influence, requesting the practitioners therein resident to transmit to the Secretary of this Society all cases of gross mal-practice that occur within their knowledge, and which admit of being duly substantiated; in order that they may be forwarded to the Society of Apothecaries, whose ready attention, and vigorous co-operation, with the views of the Association, your Committee thankfully acknowledges; and of which it has every reason to expect a continuance. Various communications have already been received, especially from Lancashire, stating the gross ignorance and palpable misconduct of persons who, without education, or learning, or study of any kind, have yet had the assurance to practise medicine. Your Committee earnestly hopes that, under the Apothecaries' Act, some example may be made of such unprincipled offenders, and that the same salutary consequences will every where result from their exposure as followed the well-known trial and conviction which took place in Staffordshire, in the year 1819.

The Committee cannot refrain here from expressing its marked approbation of the intelligence and activity which characterize the practitioners in the last-mentioned county; abundantly shewn in their correspondence with this committee, in the establishment of a medical and surgical library, and in the still increasing numbers of their members; there being no fewer than one hundred and thirty gentlemen resident in that county, who belong to this association.

The 15th resolution of the last general meeting recommended to the Committee, to take into consideration the 28th clause of the Apothecaries' Act; with a view to the removal of certain practices which prevail to the great injury of the profession, under the supposed sanction of that act.

After repeated and lengthened discussions on the subject, your

Committee regrets to state, that until the act referred to shall itself undergo the revision and amendment of which it stands so much in need, no hope can be entertained of instituting and enforcing any effectual remedy for the evils alluded to.

Nevertheless, a new act may be so framed as to meet the difficulties which arise from the ambiguity of that clause, and to restrain at least from prescribing for diseases and practising medicine, all those who, by a regular attendance on the schools of medicine, and a subsequent examination before competent authorities, shall not have proved themselves qualified for these important offices. Of a new act such a restrictive clause would undoubtedly form a prominent and even essential part.

In furtherance of the same objects, the Committee has laboured to carry into effect the 18th resolution of the last annual meeting, by the volume of transactions which, under the sanction of this meeting, the Committee especially appointed for that purpose, will shortly have the honour to lay before the public; they hope to shew that whilst the work contains valuable papers from individuals in every branch of the profession, part of its details will point out the prevalence and evil tendency of ignorance in those who practise the medical art, without having been properly instructed; and the remainder of the volume evinces the advantage of a systematic education of every medical man. Your Committee, believing that the attainment of these objects for which the Society has been formed will be greatly expedited by the periodical publication of similar volumes of transactions, and feeling proportionally anxious to circulate the present collection as generally as possible, amongst the members of the Society; therefore suggests, that such of them as may agree to subscribe for the work, shall receive copies at as nearly as possible its cost price; and that the remaining copies of the work shall be sold to the public at such a price as shall bring a profit to the Society; thus assisting in preventing a further diminution of the funds of the Society.

It is further to be observed, that as this intended volume will contain the annual report, and the existing rules and regulations of the association, with a list of its members, the considerable expense which has hitherto been incurred by the publication of all these in a detached form will hereafter be saved to the Association.

Thus occupied at its several meetings, your Committee, while it claims not the merit of extraordinary labour, yet ventures to cherish the hope that the Association will give credit to its members, for having performed with fidelity and zeal all the duties imposed on them, for having given their earliest attention to every communication and promising suggestion laid before them; for having economised to the utmost the funds of the Society; and, above all, for having, in every deliberation, and every act, steadily kept in view the ends for which they were appointed, and which all the members of this extensive association desire to promote, viz.—the amelioration of the condition of suffering mankind, and the respectability, the honour, and the welfare of enlightened and liberal practitioners of the healing art.

